# Temporal Comparison Reveals Weakened Genotype–Phenotype Association at a Major‐Effect Locus in Dworshak National Fish Hatchery Steelhead

**DOI:** 10.1111/eva.70300

**Published:** 2026-07-18

**Authors:** Audrey C. Harris, Stuart C. Willis, Shawn R. Narum, Matthew R. Campbell

**Affiliations:** ^1^ Pacific States Marine Fisheries Commission, Eagle Fish Genetics Laboratory Eagle Idaho USA; ^2^ Columbia River Inter‐Tribal Fish Commission, Hagerman Genetics Laboratory Hagerman Idaho USA; ^3^ Idaho Department of Fish and Game Eagle Fish Genetics Laboratory Eagle Idaho USA

**Keywords:** adaptive genetic variation, large‐effect loci, life history evolution, *six6*, steelhead

## Abstract

Life history diversity in salmonids contributes to population resilience, yet the mechanisms driving contemporary life history change remain difficult to disentangle from demographic and environmental processes. We evaluated long‐term changes in neutral genetic variation, adaptive haplotypes near the *six6* gene, and life history traits in the Dworshak National Fish Hatchery steelhead ( *Oncorhynchus mykiss* ) broodstock by comparing archived scale samples from the founding broodstock and subsequent returns (1969–1976) with contemporary samples (2014–2016; 2019–2022). Neutral genetic diversity and population structure remained stable through time, consistent with prior evidence that observed life history shifts were not associated with erosion of neutral genetic diversity. In contrast, we detected pronounced temporal shifts in adaptive variation near the *six6* gene on chromosome 25, including a decline in the long haplotype historically associated with extended years in the ocean and larger body size. Ocean age composition also shifted over time, with an increased proportion of 2‐ocean fish and altered age distributions within *six6* genotype classes. Although *six6* remained significantly associated with length‐at‐age, the magnitude of phenotypic differences among genotypes was substantially reduced in contemporary samples, indicating a weakening of the genotype–phenotype relationship. These results suggest that environmental conditions increasingly constrain, or otherwise alter, the expression of genetically based life history variation. Our findings demonstrate that life history change can occur without loss of neutral genetic diversity and highlight the importance of integrating genetic and demographic data to understand eco‐evolutionary responses of salmonids under changing ocean conditions.

## 1 Introduction

The mechanisms underlying why and how adaptation occurs are recurring themes in evolutionary biology. Classical evolutionary theory posits that natural selection typically operates over long periods of time through gradual selective pressures favoring specific traits (Hendry and Kinnison 1999). However, recent environmental data reveal pronounced shifts occurring within much shorter time periods, which suggest the rate of environmental change may surpass the ability of populations to adapt (Bell and Collins 2008). While there are many studies demonstrating the impacts of environmental change on population demography, the mechanisms driving adaptation to changing environmental conditions in natural populations are more difficult to quantify (Hansen et al. 2012; Merilä and Hendry 2014). Integrating historical datasets with modern genomic approaches provides a powerful framework to study how adaptation may occur over contemporary time scales (Bi et al. 2013; Therkildsen et al. 2019; Setzke et al. 2021; Raxworthy and Smith 2021; Han et al. 2025).

Rapid environmental change is especially challenging for migratory species, including anadromous salmonids whose life cycles depend on both freshwater and marine environments. Salmonids have evolved a vast array of life history strategies in response to unstable environmental conditions (e.g., Pleistocene glaciation, volcanism in the Pacific Ring of Fire; Lichatowich 1999; Waples et al. 2008). Although inherent trade‐offs exist for any individual life history strategy, the diversity of life histories within populations or species is paramount for buffering populations against stochasticity and mitigating extinction risk (i.e., “portfolio effect”; Hilborn et al. 2003; Schindler et al. 2010; Moore et al. 2014). Conserving life history diversity in the face of a changing climate helps preserve biodiversity while ensuring standing genetic variation is available for natural selection to act on (Barrett and Schluter 2008; Derry et al. 2019). In recent years, there has been substantial research interest in discovering loci of large effect that underlie life history traits in salmonids (Barson et al. 2015; Hess et al. 2016; Prince et al. 2017; Waters et al. 2021; Waples et al. 2022), but much remains unknown about how these large‐effect loci respond to changes in environmental conditions and facilitate adaptation (though see Jensen et al. 2022; Besnier et al. 2024). Despite this knowledge gap, shifts in life history characteristics of anadromous salmonids are already occurring in response to environmental change, with documented reductions in life history complexity in hatchery steelhead in California (Huber et al. 2024), younger age composition in multiple salmon species in Alaska (Oke et al. 2020), and increased abundance of odd‐year returning broodlines of pink salmon relative to even‐year broodlines, especially in the southern portion of their range (Irvine et al. 2014).

Two candidate genes that have been implicated in life history diversity across several salmonid species are the sine oculis homeobox homolog 6 (*six6*) and vestigial‐like family member 3 (*vgll3*) genes (Barson et al. 2015; Sinclair‐Waters et al. 2020). In Atlantic salmon ( *Salmo salar* ), the *six6* gene has been associated with a suite of life history characteristics, both as a singular predictor and in epistatic interactions with the *vgll3* gene. In Atlantic salmon caught at sea, *six6* was associated with stomach content weight for individuals with non‐empty stomachs across all ocean age classes and the probability of stomach fullness for 1‐ocean individuals (Aykanat et al. 2020). Both *six6* and *vgll3* were associated with functional morphological traits in Atlantic salmon reared in common garden conditions, with *vgll3* linked to traits involved in swimming performance and *six6* associated with variation in body‐head proportions (Aykanat et al. 2025). Researchers also found complex interplay between *vgll3* and *six6* genotypes and enzymatic activity in the heart and intestine, indicating that epistatic interactions of these genes may play a role in organismal metabolism (Prokkola et al. 2024).

Other work in Atlantic salmon has documented how these large‐effect loci may respond to changes in selective regimes and facilitate rapid adaptation. One study found that *vgll3* and *six6* genotypes accounted for 80% of the observed decline in Atlantic salmon body mass from 1926 to 2016 after a steep reduction in river discharge (Jensen et al. 2022). These genes also demonstrated sex‐specific epistasis in their association with Atlantic salmon age‐at‐maturity, where only males with two *vgll3* late alleles exhibited an association between age‐at‐maturity and *six6* (Besnier et al. 2024). However, a substantial reduction in the strength of association between age‐at‐maturity and *vgll3*/*six6* genotypes was observed in contemporary samples, suggesting temporal changes in the association between genotype and phenotype as a function of changing marine conditions (Besnier et al. 2024).

Although much of the research surrounding large‐effect loci and life history diversity of salmonids has focused on Atlantic salmon due to their commercial value and readily available genomic resources, rainbow trout ( *Oncorhynchus mykiss* ) and their anadromous form, steelhead, are also an effective study organism to answer questions about the interplay between life history diversity, large‐effect loci, and adaptation. Steelhead are one of the most diverse Pacific salmonids, with variation in overall life history strategy (anadromy vs. residency), reproductive strategy (semelparity vs. iteroparity), age at smoltification, age at maturity, and migration timing (Quinn 2018). Their broad geographic range, from the Kamchatka Peninsula in Russia to the western coast of North America (Whiteley et al. 2019), also demonstrates their adaptability to varied environmental conditions. In some populations, life history traits are influenced by large‐effect loci (Hess et al. 2016; Prince et al. 2017; Pearse et al. 2019; Willis et al. 2020, 2024), but effects vary by geographic location and population.

Unlike Atlantic salmon, *vgll3* has not been consistently associated with life history traits in steelhead, and age‐at‐maturity and length‐at‐age are more closely associated with a region on chromosome 25 near the *six6* gene (Waters et al. 2021; Willis et al. 2020). In the Columbia River Basin, multiple linked single nucleotide polymorphisms (SNPs) in this region are associated with age and length in steelhead adults returning to spawn and can be phased into haplotypes, often classified as “short” and “long” (Willis et al. 2020). The short haplotype is associated with shorter ocean residence and smaller body size, whereas the long haplotype is associated with longer ocean residence and larger body size. Throughout this paper, we refer to these haplotypes as *six6* haplotypes, though we acknowledge that the linked SNPs in this region correspond not only to the *six6* gene but also to variation in the intergenic region upstream, potentially reflecting regulatory variants.

Previous work that linked *six6* to duration of ocean residence and body size focused on wild Columbia River Basin steelhead, and hatchery‐ or stock‐specific analyses remain lacking. Hatchery populations can provide additional insight into associations between large‐effect loci and life history characteristics because they often afford larger sample sizes and impose partial control over early life history characteristics (e.g., fixed freshwater rearing duration), which can help disentangle confounded variables such as ocean age and length. The Dworshak National Fish Hatchery steelhead broodstock provides an intriguing case study for understanding how large‐effect loci influence life history traits. Construction of Dworshak Dam on the North Fork Clearwater River in Idaho, USA occurred between 1968 and 1973, and the Dworshak National Fish Hatchery steelhead broodstock was founded to mitigate for loss of anadromous fish passage above the dam. This broodstock originated from an anadromous population in the North Fork Clearwater River, a Snake River tributary within the Columbia River Basin. Dworshak steelhead are typically older and larger at return than other steelhead stocks in the Snake River Basin (Robards and Quinn 2002; Bowersox et al. 2019). A majority of returning adults spend 2 years in the ocean (Bowersox et al. 2019), and the stock has a higher proportion of the *six6* long haplotype than other Snake River stocks (Harris et al. 2025). Over time, managers have documented a decline in length‐at‐age in all ocean age classes, but found this decline was unrelated to loss of neutral genetic diversity (Bowersox et al. 2019). However, temporal shifts in *six6* haplotype frequencies have not been evaluated. Archived scale samples from the broodstock founders and early returns (1969–1976) paired with contemporary samples provide a unique opportunity to understand the timescale adaptive changes may occur on and the mechanisms that underlie them. Our primary objectives in this study were to (i) assess if *six6* haplotype frequencies have changed since the broodstock was founded and (ii) quantify the relationship between *six6* genotypes, ocean age, and length in both historical and contemporary samples.

## 2 Methods

### 2.1 Historical and Contemporary Sample Collection

In the Snake River Basin, returning adult steelhead continue to mature after returning to freshwater and are classified into spawn years, defined as the fall of the previous calendar year and the spring of the following calendar year (e.g., spawn year 1969 refers to all adult steelhead that migrated to the Snake River Basin between July 1, 1968 and June 30, 1969 and spawned in the spring of 1969). The Dworshak National Fish Hatchery broodstock was founded from wild fish during the initial construction of Dworshak Dam on the North Fork Clearwater River during spawn year 1969. During broodstock collection in spawn years 1969–1976, hatchery personnel collected and mounted one to three scales on gum cards from a subset of adult spawners and recorded biological data, including phenotypic sex, length, and weight. Hatchery staff also created acetate impressions of scale gum cards using a heated hydraulic press. Scale cards, acetate impressions, and data sheets were archived until 2021, when data sheets were digitized, acetate impressions and scale cards were aged at the Nampa Research Anadromous Ageing Laboratory (NRAAL), and scale cards were sent to the Eagle Fish Genetics Lab for genotyping (*n* = 3871).

Fin clips from all Dworshak National Fish Hatchery broodstock have been collected annually by hatchery personnel as part of the Snake Basin parentage‐based tagging (PBT) program since 2008 (Steele et al. 2019; Hargrove et al. 2025), and we used samples from spawn years 2014–2016 and 2019–2022 as the contemporary group for this study (*n* = 11,229). The neutral genetic diversity of spawn years 2014–2016 was previously compared to the founding broodstock from 1969 (Bowersox et al. 2019), and these contemporary years were included in the present study to allow direct comparisons to prior work. All fin clips were stored on sheets of Whatman chromatography paper (LaHood et al. 2008), and biological data collected alongside contemporary samples included length and phenotypic sex. It is important to note that some level of artificial selection occurs at the hatchery, and managers have prioritized spawning larger fish when return sizes are large enough, especially in recent years (IDFG, NPT, and USFWS 2022). This practice could bias spawned broodstock toward larger body size and, if *six6* remains associated with body size, toward associated haplotypes. Because only spawned broodstock are sampled and genotyped, our results are influenced not only by the characteristics of the full adult return, but also by hatchery collection and spawning practices (see Discussion).

### 2.2 Laboratory Methods, Genotyping, and Filtering

To prepare historical samples, we carefully removed all available scales from the squares of each gum card or microscope slide and transferred them to 96‐well plates for DNA extraction. For each contemporary sample, a 3‐mm^2^ piece of fin tissue was transferred to a 96‐well plate for DNA extraction. We extracted DNA from scales and fin tissue using nondenatured Chelex (Sigma‐Aldrich, St. Louis, Missouri) or the nexttec Genomic DNA Isolation Kit (XpressBio, Thurmont, Maryland) according to the manufacturer's protocol. A panel of 368 SNPs was amplified via genotyping‐in‐thousands by sequencing (GT‐seq; Campbell et al. 2015; full laboratory protocol available at https://doi.org/10.17504/protocols.io.j8nlko1e6v5r/v1), which uses unique combinations of plate‐ and well‐specific barcodes to enable the pooling of samples for sequencing and assignment of sequenced DNA fragments back to individual samples. The SNP panel contained 242 neutral loci, 122 putatively adaptive loci (including 10 loci on Chromosome 25 in or near *six6*), three species diagnostic markers, and a genetic sex marker. Pooled libraries were sequenced on a Nextseq 500 (Illumina, San Diego, California), and a custom bioinformatics pipeline was used to assign sequencing reads back to individual samples, determine ploidy, assess contamination, and call genotypes. We determined ploidy based on read counts using the methods described in Delomas (2019) and implemented in the tripsAndDip R package. Contamination was assessed using the individual fuzziness index (IFI) score, which uses read counts at homozygous and no‐call loci to evaluate background noise (Campbell 2017).

We first removed individuals with < 90% genotyping success at all SNPs, IFI scores > 3.2, and triploid ploidy calls to ensure high genotype quality. We then performed a duplicate search requiring 70% non‐missing genotypes and 95% shared genotypes with the close_matching_samples function from the rubias R package (Moran and Anderson 2019). For each set of genetic duplicates, a single individual with the highest overall genotyping success was retained. After searching for duplicates, we removed individuals with discordant genetic and phenotypic sex and < 90% genotyping success at the 10 Chromosome 25 SNPs. Unless specified otherwise, all analyses were performed in R version 4.2.2 (R Core Team 2022), and all plots were created using the ggplot2 R package (Wickham 2016).

### 2.3 Age Determination

In fishes, annual growth rings on scales, known as annuli, can be counted to determine age (Quist et al. 2012). Changes in growth between freshwater and marine phases in steelhead allow differentiation of annuli deposited during each period, and total age can be divided into freshwater and ocean age components (Reinhardt et al. 2022). To determine ages for historical scale samples, personnel at the NRAAL recorded digital images of scales using a compound microscope with an attached camera (Wright et al. 2015). Because the acetate impressions had warped over time, a jig crafted from two microscope slides taped together at one end was used to anchor the acetate to the microscope stage and flatten the impressions so the entire scale was in focus during imaging. If the rough surface of scales was originally placed face down on the gum cards, the corresponding acetate impression was smooth and un‐ageable. In these cases, NRAAL personnel removed scales from the gum cards, mounted them between microscope slides, and imaged them. Scales mounted between slides were not cleaned to preserve any remaining tissue for genetic analysis. Ages were recorded using European notation, where age is represented by two numerals separated by a period. The first numeral corresponds to freshwater age, and the second numeral corresponds to ocean age. Total age was calculated by adding the freshwater age, ocean age, and one to account for the winter spent in freshwater before spawning (Wright et al. 2015). Accuracy and precision of scale ages at the NRAAL are validated using systematic quality assurance and control measures (Copeland et al. 2018; Reinhardt et al. 2022; Davison et al. 2024).

Contemporary samples were aged via genetic pedigree reconstruction as part of the Snake River Basin PBT program (Steele et al. 2013, 2019). For contemporary samples with parentage assignments, total age was derived by subtracting the spawn year of the assigned parent pair from the spawn year of the offspring. Because all Dworshak National Fish Hatchery steelhead are reared for 1 year before being released as smolts, all contemporary samples had a freshwater age of one. Ocean age was calculated by subtracting the freshwater age (1) and time spent overwintering in freshwater before spawning (1) from the total age.

### 2.4 Neutral Genetic Structure

We evaluated evidence of neutral genetic population structure among spawn years using a discriminant analysis of principal components (DAPC), which first reduces dimensionality using a principal components (PCs) analysis and then applies discriminant functions (DFs) to minimize within‐group variance and maximize between‐group variance (Jombart et al. 2010). Because we were interested in neutral genetic structure, we performed the DAPC using only the 242 putatively neutral SNPs in the GTseq panel. Before fitting the DAPC, we used PLINK 1.9 (Purcell et al. 2007) to assess linkage between markers and retained a single marker from each pair of markers with *R* ^2^ > 0.2. DAPC can be sensitive to the number of retained PCs; we followed the recommendations of Thia (2023) and retained K−1 PCs, where K was equal to the number of spawn years in the dataset. Each spawn year was used as an a priori cluster for DAPC, and we retained all DFs.

### 2.5 Phasing Haplotypes and Assessing Temporal Shifts

We used the haplo.em function from the haplo.stats R package (Sinnwell and Schaid 2024) to phase chromosome 25 SNPs into *six6* haplotypes separately for each spawn year. The haplo.em function generates haplotype calls using an expectation–maximization algorithm that progressively inserts loci in batches, trims haplotype pairs below a certain probability threshold, and then continues the insertion and trimming steps until all loci have been added (Sinnwell and Schaid 2024). We specified a batch size of six loci for progressive insertion, 100 attempts for haplotype estimation, and default parameters for all other options. For each individual, we retained the pair of *six6* haplotypes (i.e., *six6* genotype) with the highest posterior probability.

Based on the findings of Willis et al. (2020), which examined haplotype frequencies and associations in steelhead across the Columbia River Basin, *six6* haplotypes were categorized into three classes: short (S), long (L), and other (i.e., any haplotype not designated as short or long). Each individual's *six6* genotype was classified as short homozygote (SS: two copies of the short haplotype), heterozygote (SL: one copy of the short haplotype and one copy of the long haplotype), long homozygote (LL: two copies of the long haplotype), or other (at least one copy of a haplotype that was neither short nor long). To assess whether temporal shifts in haplotype frequencies occurred since the broodstock was founded, 100,000 bootstrap replicates were created by resampling haplotypes with replacement up to the total number of called haplotypes for each year (2*n*). Within each year, we calculated haplotype frequencies for each bootstrap replicate and constructed 95% confidence intervals for frequencies of each haplotype category using all bootstrap replicates. If confidence intervals from a given year did not overlap confidence intervals from the 1969 founding stock, haplotype frequencies for that year were considered significantly different.

To test for potential differences in ocean age composition between historical and contemporary time periods, we used a chi‐squared test on a contingency table of individuals in each age class. Fisher's exact tests were used on contingency tables of individuals in each age class within genotype categories to test if ocean age composition differed within each genotype across time periods. For all chi‐squared and Fisher's exact tests, 3‐ocean and 4‐ocean individuals were combined into a single category (i.e., ocean age 3+) due to low cell counts, and counts of age classes were pooled across year and sex within each time period, since not all age classes appeared in each year × sex combination.

### 2.6 Relationships Between Length and Haplotypes

We aimed to quantify the relationship between length and *six6* genotype while accounting for other covariates associated with length, including year, ocean age, and sex. Because haplotypes in the other category were not associated with body size or ocean age (Willis et al. 2020) and occurred in low frequency in our dataset, individuals with at least one copy of the other haplotype were excluded from our modeling efforts. We also excluded individuals with missing data for length or ocean age, along with individuals that had an ocean age of 4, as this category occurred in very low frequency (*n* = 5). Like many populations of steelhead, the Dworshak National Fish Hatchery broodstock exhibits a female‐biased sex ratio (Copeland et al. 2017), and this pattern is particularly prevalent in historical samples (Figure S1). To assess the impact of smaller sample sizes for males on our modeling inference, we repeated all modeling methods detailed below on a dataset of only females and presented results in Supporting Information.

Sum contrast options were specified in R before fitting models to enable unbiased Type III ANOVA *F*‐tests. For our global model, we fit a linear model with length as the response variable and ocean age, sex, year, and *six6* genotype as predictor variables. We also included two‐way interaction terms between *six6* genotype and all other covariates (ocean age, year, and sex) to assess whether the relationship between *six6* genotype and length was mediated by other variables. All numeric covariates (i.e., ocean age and year) were treated as factors. We performed all‐subsets model selection on our global model using the dredge function from the MuMIn R package (Bartoń 2023) and ranked models by Akaike Information Criterion corrected for small sample sizes (AIC_c_). To determine the best‐fit model, we considered any model with ΔAIC_c_ ≤ 2 and selected the model with the fewest parameters (Burnham and Anderson 2002).

We checked for violations of linear regression assumptions in the best‐fit model using diagnostic plots and evaluated significant differences among group means by fitting a Type III ANOVA with the car R package (Fox and Weisberg 2019). After selecting the best‐fit model, estimated marginal means for all predictors were calculated using the emmeans R package (Lenth 2024). We also performed post hoc testing of differences between the predicted mean length for *six6* genotypes within each year, while controlling for sex and ocean age with the emmeans package. To determine if differences in length among genotypes were significant within years, we used Tukey‐corrected *p*‐values for multiple comparisons and considered *p* < 0.05 significant.

## 3 Results

### 3.1 Genotyping and Filtering

Across all years, 15,100 samples were genotyped, and 5873 samples did not pass our quality filters, resulting in a total of 9227 samples retained for analyses of neutral genetic structure and haplotype association (Table S1). We used 8025 samples for our model after removing individuals with missing data and those in rare categories (Table S1). Sex ratios were biased toward females, especially in historical samples; this trend intensified as individuals were excluded based on genotyping success and missing data (Figure S1). However, analyses restricted to only females produced results consistent with those from the full dataset (Figures S2–S6; Table S4). Therefore, we present results from the full dataset here.

### 3.2 Temporal Shifts in Neutral and Adaptive Markers

**FIGURE 1:**
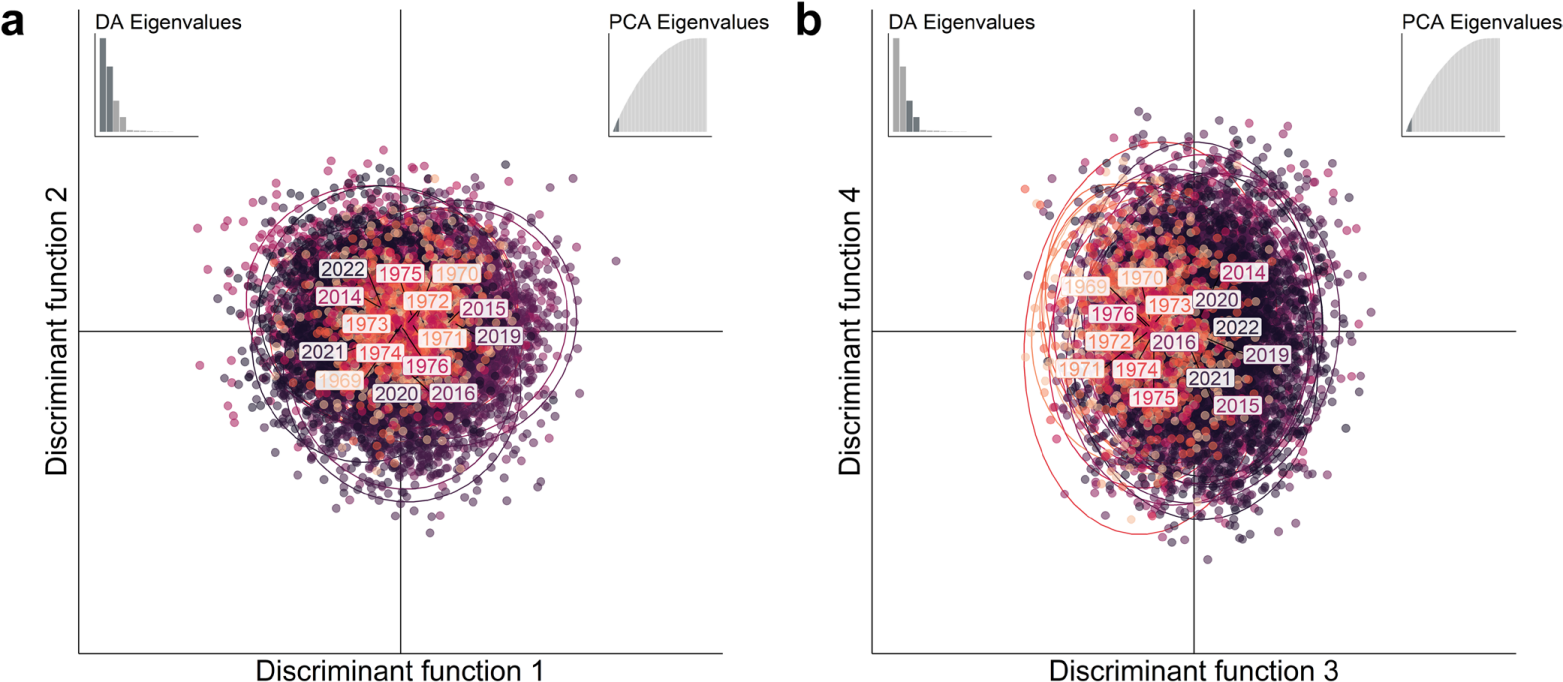
Discriminant analysis of principal components (DAPC) of neutral loci of Dworshak National Fish Hatchery steelhead broodstocks sampled across historical (1969–1976) and contemporary (2014–2016; 2019–2022) time periods using spawn years as a priori genetic clusters. Labels denote the centroid of each spawn year, each point represents an individual, and inertia ellipses represent 95% confidence intervals. Colors of points, labels, and ellipses correspond to spawn years. (a) DAPC plotted on axes of discriminant function 1 and discriminant function 2. (b) DAPC plotted on axes of discriminant function 3 and discriminant function 4.

Within the 242 neutral markers, there was a single pair with *R* ^2^ > 0.2, and we removed one of the linked pair, leaving 241 putatively neutral markers for the DAPC. We retained 14 PCs for the DAPC of neutral markers, which conserved 14.74% of the observed variance. DFs 1 and 2 explained the most variance among spawn years (DF 1 = 44.69%; DF 2 = 31.19%), and individuals from all spawn years formed a single cluster of points when plotted along DFs 1 and 2 (Figure 1a). No additional genetic structure was present when individuals were plotted along DFs 3 and 4 (Figure 1b), which explained less variance (DF 3 = 14.76%; DF 4 = 7.04%). Our DAPC results suggest no temporal differences between historical and contemporary collections at neutral markers.

**FIGURE 2:**
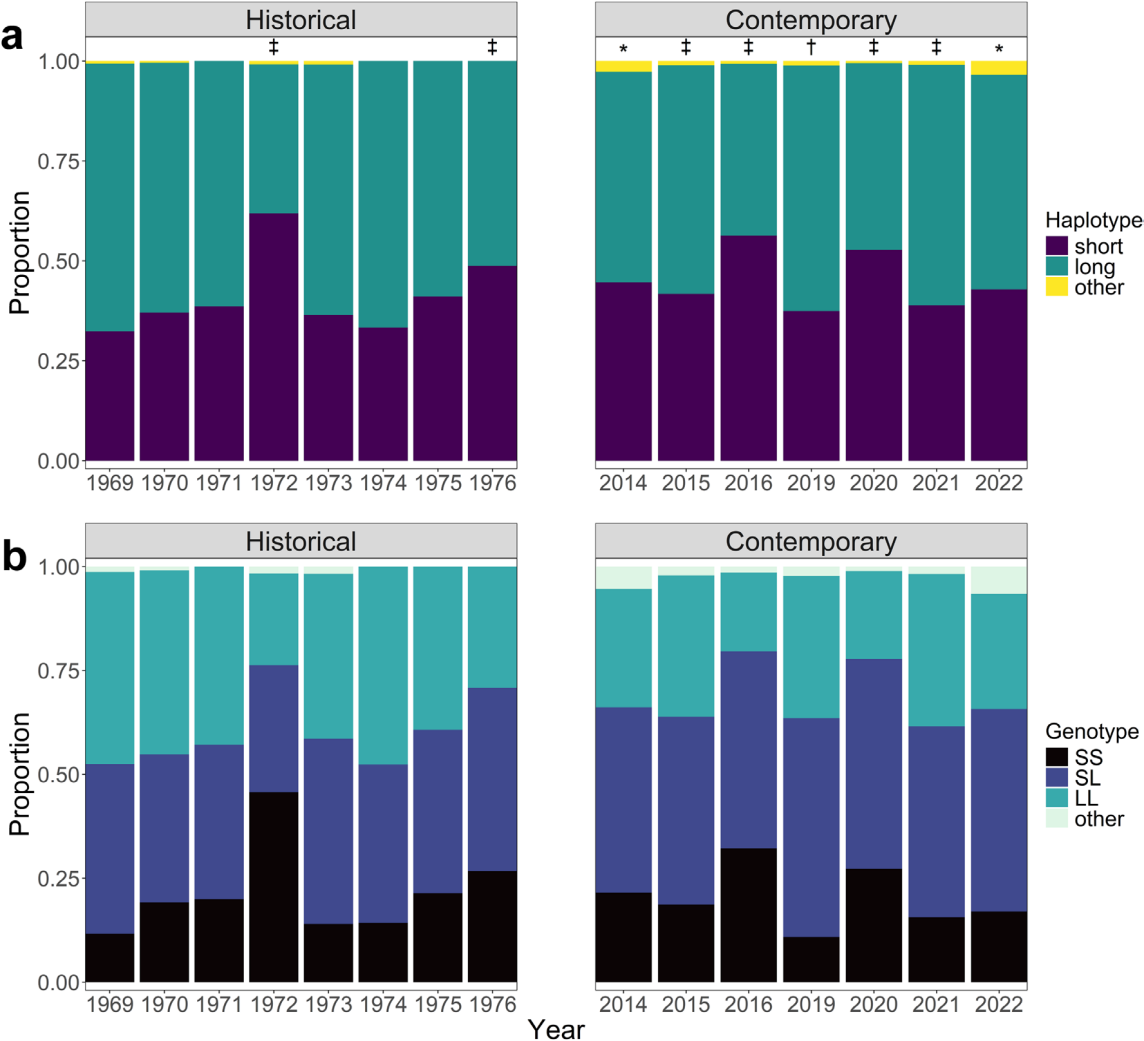
*Six6* haplotype and genotype frequencies of Dworshak National Fish Hatchery steelhead broodstocks across historical (1969–1976) and contemporary (2014–2016; 2019–2022) time periods. (a) *Six6* haplotype frequencies by year. Years with statistically significant differences in haplotype frequencies from 1969 are denoted above the bars—† represents differences in only the long haplotype, ‡ denotes differences in both long and short haplotypes, and * signifies differences in short, long, and other haplotypes. (b) *Six6* genotype frequencies by year.

Across all years, 15 unique haplotypes were detected (Table S2), though the long and short haplotypes first described in Willis et al. (2020) were most common. The long haplotype was the most frequently observed haplotype in all years except 1972, 2016, and 2020 (Figure 2a). In all contemporary years, the most common genotype was heterozygous (SL), whereas the long homozygous (LL) genotype was most frequent in five of the nine historical years (Figure 2b). Compared to baseline frequencies in 1969, we detected significant shifts in haplotype frequencies in 1972, 1976, and all contemporary years (Figure 2a). In most years with significant deviations from 1969, frequencies of both the long and short haplotypes contributed to the shift; however, in 2019, only the change in long haplotype frequency was significant (Figure 2a).

**FIGURE 3:**
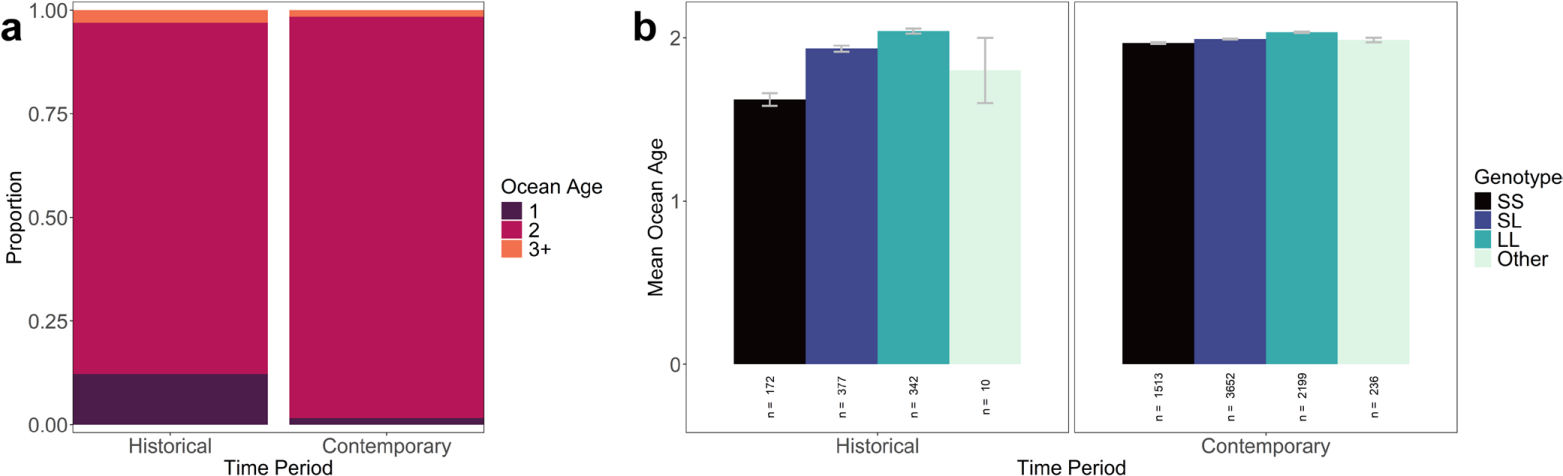
Overall ocean age composition and mean ocean age by *six6* genotype of Dworshak National Fish Hatchery steelhead broodstocks across historical (1969–1976) and contemporary (2014–2016; 2019–2022) time periods. (a) Proportion of individuals in each ocean age class. (b) Mean ocean age by *six6* genotype class. Error bars represent standard error.

Overall ocean age composition differed significantly between historical and contemporary samples (*χ* ^2^ = 360.86, df = 2, *p* = 4.37 × 10^−79^), likely driven by a greater proportion of 2‐ocean adults and a decline in the proportion of 1‐ocean adults in contemporary samples (Figure 3a). Within each genotype category, ocean age composition also differed significantly between time periods, as indicated by Fisher's exact tests (SS *p* = 1.60 × 10^−39^; SL *p* = 6.01 × 10^−15^; LL *p* = 1.06 × 10^−4^; other *p* = 0.002). In addition, mean ocean age increased for individuals with SS and SL genotypes in contemporary samples (Figure 3b), demonstrating a weakening of the association between genotype and ocean age over time.

### 3.3 Relationships Between Length and Haplotypes

**FIGURE 4:**
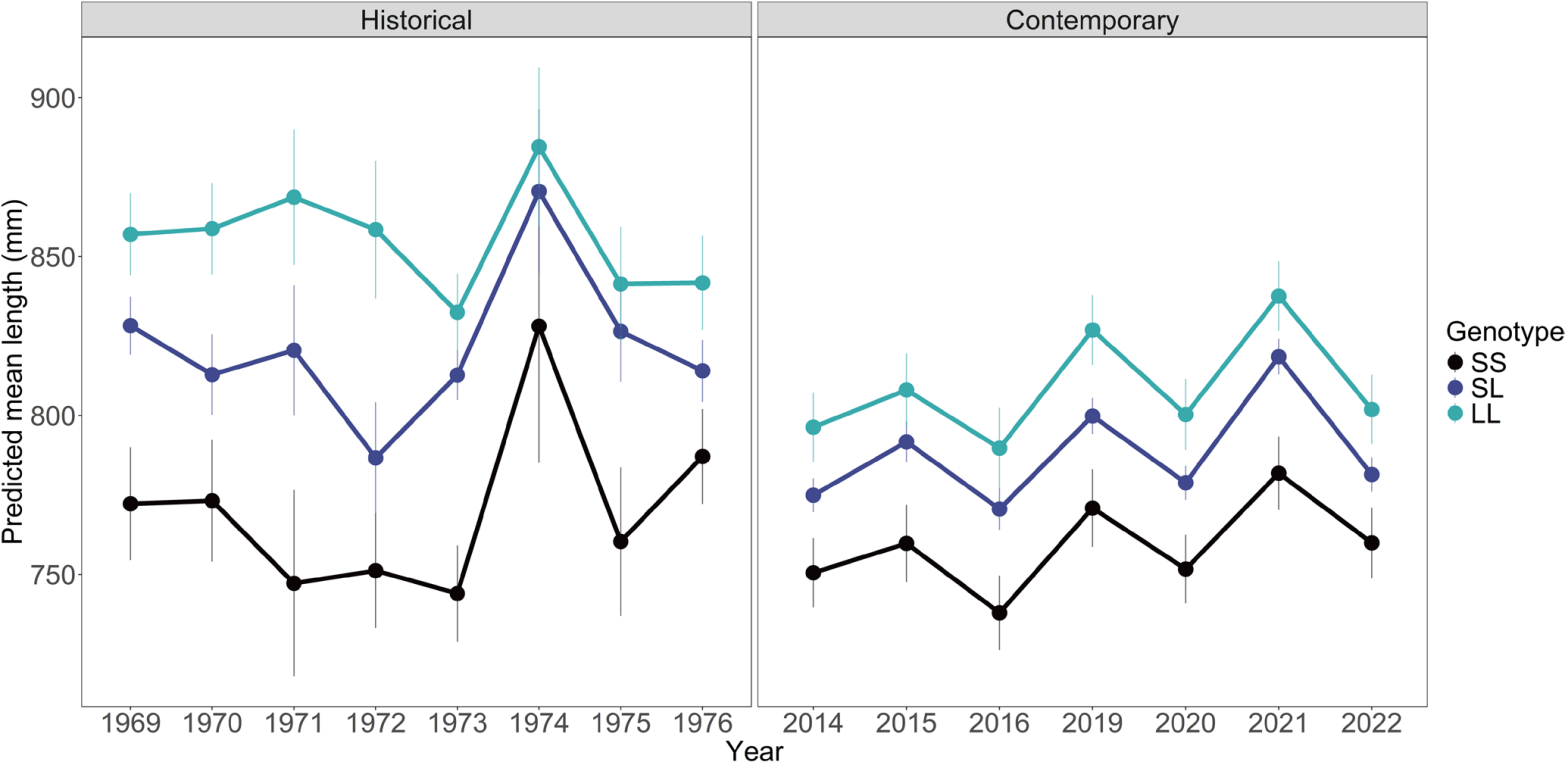
Predicted mean length for each *six6* genotype across historical (1969–1976) and contemporary (2014–2016; 2019–2022) time periods. Error bars represent 95% confidence intervals for each estimated marginal mean.

The best‐fit model was our global model (AIC_c_ = 80703.736), which included terms for ocean age, sex, year, and *six6* genotype, with two‐way interaction terms between *six6* genotype and ocean age, sex, and year. There were no competing models within ΔAIC_c_ ≤ 2. The adjusted *R* ^2^ for the best‐fit model was 0.522, and all terms in the model were statistically significant based on Type III ANOVA F‐tests (*p* < 0.001 for all variables; Table S3). Taken together, these results demonstrate that our model explained a substantial proportion of variation in body length and that each term explained significant variance after accounting for effects of other covariates. Estimated marginal means indicated a clear relationship between *six6* genotype and body size. Even after controlling for sex and ocean age, the SS genotype was associated with smaller body size, the LL genotype was associated with larger body size, and heterozygotes (SL) exhibited intermediate body size (Figure 4).

**FIGURE 5:**
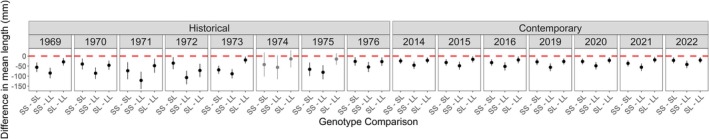
Contrasts of predicted length between genotype categories within each year. Error bars represent 95% confidence intervals for each contrast, and statistical significance is denoted by color. Zero is denoted by the dashed red line. Contrasts that significantly differ from zero are black, and contrasts that overlap with zero are gray.

**FIGURE 6:**
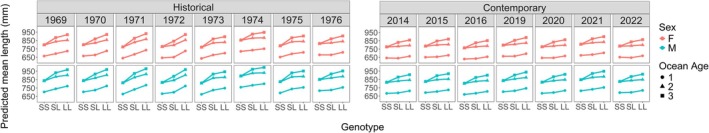
Predicted mean length for each combination of *six6* genotype, sex, and ocean age across historical (1969–1976) and contemporary (2014–2016; 2019–2022) time periods.

Post hoc testing of differences in predicted length between genotype categories within years indicated that most contrasts were significant, with the exception of a few contrasts in 1974 and 1975 (Figure 5). Across all years, the largest differences in mean length consistently occurred between homozygous genotypes (i.e., SS−LL). In contrast, comparisons involving heterozygotes (i.e., SS−SL and SL−LL) demonstrated inconsistent rankings among years (Figure 5). Notably, the strength of the association between *six6* genotype and body length diminished over time, as evidenced by much smaller, albeit still significant, differences in predicted length among genotypes in contemporary years (Figure 4; Figure 5). This temporal attenuation is further illustrated by a general decline in predicted mean length across all sex × ocean age combinations in contemporary years relative to historical years (Figure 6).

## 4 Discussion

Our results add to a growing body of work showing that large‐effect loci associated with key life history traits can respond rapidly to changing selective regimes over contemporary timescales (Jensen et al. 2022; Besnier et al. 2024). Although demographic changes have been documented in the Dworshak steelhead broodstock (Bowersox et al. 2019), we found that genetic population structure at neutral markers remained stable between historical and contemporary samples. Consistent with earlier work, this suggests that the observed life history shifts are not explained by erosion of neutral genetic diversity or significant immigration from other stocks (Bowersox et al. 2019). Similar temporal stability at neutral markers has been reported in some long‐term salmonid genetic monitoring studies, reinforcing that neutral stability does not preclude demographic shifts or changes at adaptive loci (Van Doornik et al. 2011).

If the patterns observed were due to size‐selective spawning practices implemented at Dworshak National Fish Hatchery, where larger steelhead are preferentially spawned (IDFG, NPT, and USFWS 2022), we would expect spawned broodstock to be biased toward larger fish and, assuming the historical *six6*‐body size association still holds, toward higher long haplotype frequencies. In contrast, frequencies of the *six6* long haplotype were substantially lower in contemporary broodstocks compared to the founding broodstock from 1969. Therefore, preferential spawning of larger fish alone is unlikely to explain the observed decline in the long haplotype; if anything, this practice could make the decline appear smaller in spawned broodstock than it would in the full adult return. Ocean age composition, both overall and within *six6* genotype category (SS, SL, LL), also significantly differed between time periods, and mean ocean age increased for contemporary individuals with SS and SL genotypes. Although *six6* genotypes were associated with length‐at‐age in both historical and contemporary years, the magnitude of differences in length between genotypes was greatly reduced in the contemporary time period, even after accounting for sex and ocean age.

These patterns are consistent with well‐documented shifts in age composition and size structure across multiple species of anadromous salmonids (Ricker 1981; Ohlberger et al. 2018, 2023, 2025; Bowersox et al. 2019; Oke et al. 2020; Vollset et al. 2022). Observed changes in salmonid age and size structure have been attributed to multiple, interacting ecosystem drivers during the marine phase, encompassing climate variation and competitive interactions (Oke et al. 2020; Vollset et al. 2022; Ohlberger et al. 2023, 2025; Vosbigian et al. 2024). Our findings also parallel recent work in Atlantic salmon demonstrating a substantial reduction in the explanatory power of *vgll3* and *six6* for age‐at‐maturity between historical and modern collections (Besnier et al. 2024). Significant reductions in the frequency of the *six6* long haplotype, accompanied by weakened associations with ocean age and length, suggest contributions from both selection (i.e., differential survival or reproductive success among genotypes) and environmentally mediated plasticity (i.e., genotype‐by‐environment effects on trait expression; Merilä and Hendry 2014). Because many potential causative mechanisms interact in complex ways and operate at different scales, it is challenging to pinpoint individual factors that contributed to the observations in this study, especially given that broodstock characteristics can also be influenced by collection and spawning practices. Nevertheless, we highlight two non‐exclusive possibilities in the following paragraphs.

First, increases in sea surface temperature can reduce marine survival of steelhead (Ohlberger et al. 2025) and intensify metabolic demands for ectotherms, which may constrain growth opportunities (Gardner et al. 2011) and potentially influence age‐at‐maturity by modifying growth thresholds at which fish mature (Thorpe 2007; Besnier et al. 2024; Burton et al. 2025). Many other studies point to increasing pink salmon abundance and the complex impacts on North Pacific food webs (Ruggerone and Nielsen 2004; Springer and van Vliet 2014; Ruggerone et al. 2023), including direct and indirect influences on steelhead growth and survival (Ohlberger et al. 2025; Vosbigian et al. 2024). We observed odd‐even year fluctuations in predicted mean length across all *six6* genotypes (SS, SL, LL) in contemporary years (Figure 4), which may correspond with the cyclic nature of pink salmon abundance. However, mean differences between *six6* genotypes did not differ among even and odd years (Figure 5), suggesting that while predicted lengths may be reduced in years when pink salmon abundance is high, the association of *six6* genotype and length remains similar between odd and even years in contemporary samples.

Second, physiological and molecular mechanisms could contribute to the temporal weakening of the genotype–phenotype association we observed, but much remains unknown about the processes through which *six6* influences body size in steelhead and the relative influence of environmental conditions on phenotypic expression. Phenotypic plasticity can arise through pathways that are reciprocally interconnected yet operate at different biological scales, including functional traits at the organismal level, cellular responses within organs and tissues, and regulation of gene expression at the molecular level (Brun Usan et al. 2025). In Atlantic salmon, epistasis of *vgll3* and *six6* has been linked to tissue‐specific variation in enzymatic activity and whole organism metabolism (Prokkola et al. 2024), functional morphology and resource acquisition (Aykanat et al. 2020, 2025), and life history characteristics (Barson et al. 2015; Besnier et al. 2024). In Columbia River steelhead, SNPs upstream of *six6* show stronger associations with length and ocean age than SNPs within the *six6* coding region, suggesting a potential regulatory role (Willis et al. 2020). In addition, loci associated with iteroparous spawning phenology in female steelhead show elevated linkage disequilibrium with *six6*, indicating that this region may influence multiple components of life history via pleiotropy (Willis et al. 2024). Future work explicitly linking *six6* genotypes to phenotypes related to physiology, growth, and maturation at multiple biological scales across environmental gradients will help identify the mechanisms that govern context‐dependent expression of life history traits in steelhead.

Our findings suggest that selection for larger broodstock does not necessarily translate into a greater proportion of large adults because hatchery selection occurs only after adults have survived to return, and environmental conditions in the ocean may impose opposing selective pressures. Though the intention behind selectively breeding larger fish is to increase the proportion of large fish in the population for the benefit of anglers, this practice may be insufficient to produce the intended phenotypic response during periods when ocean conditions are less hospitable to large fish (i.e., periods of elevated sea surface temperature and low prey abundance). Compared to smaller fish, larger fish exhibit higher standard metabolic rates at a given temperature, and increased temperatures further escalate the already high metabolic demands of large fish (Clarke and Johnston 1999; Killen et al. 2010; Chabot et al. 2016). In years when sea surface temperatures are high and prey availability is low, large fish may be unable to meet their increased metabolic demands, potentially resulting in reduced condition, growth, and survival relative to smaller conspecifics (Pörtner and Knust 2007; Ohlberger 2013; Peralta‐Maraver and Rezende 2021; Lindmark et al. 2022). These ocean‐mediated effects could reduce the number or condition of large adults available for hatchery spawning before intentional broodstock selection occurs. However, the evolutionary effects of this practice in Dworshak National Fish Hatchery steelhead are difficult to quantify from spawned broodstock alone and may be self‐limiting because opportunities for size‐selective spawning increase with adult return abundance, and managers are only able to selectively spawn large fish when there is a surplus of available spawners. Consequently, environmental conditions that reduce adult returns may also limit the strength of hatchery‐imposed selection for larger body size. Future monitoring that involves genotyping both spawned and unspawned adults would allow direct estimation of the selection imposed by broodstock choice.

The Dworshak broodstock has experienced a measurable reduction in several components of life history diversity since 1969, evidenced by a decline in the *six6* long haplotype, along with an increased proportion of 2‐ocean adults and a reduction in length‐at‐age in contemporary samples, regardless of *six6* genotype. Although neutral genetic structure remained stable, the magnitude of phenotypic differentiation among *six6* genotypes has diminished, and ocean age composition has become more homogenized among genotypes through time. Together, these patterns indicate that contemporary broodstocks exhibit less functional life history variation than historical collections. Life history variation in salmonids is integral to long‐term persistence, and this narrowing of demographic and phenotypic diversity may weaken portfolio effects and increase susceptibility to environmental variability (Hilborn et al. 2003; Schindler et al. 2010; Moore et al. 2014). With increased proportions of 2‐ocean fish, fewer brood years contribute to each spawn year, resulting in reduced overlap among cohorts and larger potential impacts of stochastic environmental conditions (e.g., year‐class failures). Because life history diversity and cohort overlap are central mechanisms underlying portfolio effects, this compression of brood year contributions may reduce demographic buffering against environmental variability. In addition, reductions in body size across all ocean age classes likely decrease fecundity, as egg production in salmonids scales positively with female body length (Healey and Heard 1984; Quinn et al. 2011). Even if ocean age remains variable, smaller size‐at‐age may reduce demographic resilience across all genotype classes due to reductions in fecundity (Oke et al. 2020).

Our results further demonstrate that the strength and predictive power of genotype–phenotype associations at large‐effect loci can change over time. While large‐effect loci may be sensitive to changing environmental conditions, facilitate rapid adaptation, and influence adaptive potential, our findings reinforce that static relationships cannot be assumed between phenotype and large‐effect loci over time, and under sufficiently strong environmental constraint, the contribution of a large‐effect locus may become small relative to other sources of phenotypic variation (Besnier et al. 2024). Therefore, management and conservation strategies that rely on maintaining specific alleles or phenotypes (e.g., targeted broodstock selection) should evaluate phenotypic outcomes across a range of environmental contexts to assess how relationships between genotype and phenotype may shift (Hansen et al. 2012; Merilä and Hendry 2014; Derry et al. 2019).

## 5 Conclusions

Integrating historical samples from Dworshak National Fish Hatchery steelhead broodstock founders and early returns (1969–1976) with contemporary samples, we showed that (i) *six6* haplotype frequencies differed markedly between historical and contemporary time periods while population structure at neutral markers remained stable, and (ii) associations of *six6* genotype with both ocean age and length‐at‐age have weakened over time. This study highlights the utility of long‐term sample archives, genetic monitoring programs, and hatchery broodstocks for evaluating demographic, genetic, and environmental contributions to life history shifts in salmonid populations.

Overall, our findings indicate that maintenance of standing genetic variation alone may not preserve life history diversity if environmental conditions constrain or alter associations between genetically based life history variation and phenotype. Future work to predict evolutionary responses of salmonids to changing selective regimes will require disentangling the relative roles of selection and environmentally mediated plasticity across multiple life stages, as well as identifying the mechanisms underlying the dissociation of genotype and phenotype.

## Funding

This work was funded by Bonneville Power Administration grant number 2010‐031‐00.

## Ethics Statement

Fish were handled in accordance with the American Fisheries Society guidelines for fish collection and sampling.

## Conflicts of Interest

The authors declare no conflicts of interest.

## Supporting information


**Figure S1:** Sex ratio of all collected samples, samples used for haplotype analyses, and samples used for modeling in each spawn year.
**Figure S2:** *Six6* haplotype and genotype frequencies of females in Dworshak National Fish Hatchery steelhead broodstocks across historical (1969–1976) and contemporary (2014–2016; 2019–2022) time periods. (a) *Six6* haplotype frequencies by year. Years with statistically significant differences in haplotype frequencies from 1969 are denoted above the bars—† represents differences in only the long haplotype, ‡ denotes differences in both long and short haplotypes, and * signifies differences in short, long, and other haplotypes. (b) *Six6* genotype frequencies by year.
**Figure S3:** Overall ocean age composition and mean ocean age by *six6* genotype of females in Dworshak National Fish Hatchery steelhead broodstocks across historical (1969–1976) and contemporary (2014–2016; 2019–2022) time periods. (a) Proportion of individuals in each ocean age class. (b) Mean ocean age by *six6* genotype class. Error bars represent standard error.
**Figure S4:** Predicted mean length for females of each *six6* genotype across historical (1969–1976) and contemporary (2014–2016; 2019–2022) time periods. Error bars represent 95% confidence intervals for each estimated marginal mean.
**Figure S5:** Contrasts of predicted length for females between genotype categories within each year. Error bars represent 95% confidence intervals for each contrast, and statistical significance is denoted by color. Zero is denoted by the dashed red line. Contrasts that significantly differ from zero are black, and contrasts that overlap with zero are gray.
**Figure S6:** Predicted mean length for females of each combination of *six6* genotype and ocean age across historical (1969–1976) and contemporary (2014–2016; 2019–2022) time periods.

**Table S1:** Samples genotyped and used for analyses, broken down by the numbers remaining after each quality filtering step.
**Table S2:** Unique *six6* haplotypes in historical (1969–1976) and contemporary (2014–2016; 2019–2022) time periods, with frequencies of haplotypes in each year.
**Table S3:** Type III ANOVA F‐test results for each factor included in the best‐fit model.
**Table S4:** Type III ANOVA F‐test results for each factor included in the female‐only best‐fit model.

## Data Availability

Data for this study are available to download from fishgen.net, a repository for fisheries genetic data. The dataset can be found under Data Sets>Export/Edit>Search Options by searching for ‘Historic and contemporary Dworshak steelhead broodstocks (1969‐1976, 2014‐2016, 2019‐2022)’ (Dataset ID: 20260351). Users must create a username and log into the FishGen website before downloading data.
