# Supplementary material for: Temporal Comparison Reveals Weakened Genotype–Phenotype Association at a Major‐Effect Locus in Dworshak National Fish Hatchery Steelhead
Source: Evol Appl. 2026 Jul 18;19(7):e70300. doi: 10.1111/eva.70300 (PMC13379774; doi:10.1111/eva.70300)
Supplement: Supplementary file 1 — Figure S1: Sex ratio of all collected samples, samples used for haplotype analyses, and samples used for modeling in each spawn year. Figure S2: Six6 haplotype and genotype frequencies of females in Dworshak National Fish Hatchery steelhead broodstocks across historical (1969–1976) and contemporary (2014–2016; 2019–2022) time periods. (a) Six6 haplotype frequencies by year. Years with statistically significant differences in haplotype frequencies from 1969 are denoted above the bars—† represents differences in only the long haplotype, ‡ denotes differences in both long and short haplotypes, and * signifies differences in short, long, and other haplotypes. (b) Six6 genotype frequencies by year. Figure S3: Overall ocean age composition and mean ocean age by six6 genotype of females in Dworshak National Fish Hatchery steelhead broodstocks across historical (1969–1976) and contemporary (2014–2016; 2019–2022) time periods. (a) Proportion of individuals in each ocean age class. (b) Mean ocean age by six6 genotype class. Error bars represent standard error. Figure S4: Predicted mean length for females of each six6 genotype across historical (1969–1976) and contemporary (2014–2016; 2019–2022) time periods. Error bars represent 95% confidence intervals for each estimated marginal mean. Figure S5: Contrasts of predicted length for females between genotype categories within each year. Error bars represent 95% confidence intervals for each contrast, and statistical significance is denoted by color. Zero is denoted by the dashed red line. Contrasts that significantly differ from zero are black, and contrasts that overlap with zero are gray. Figure S6: Predicted mean length for females of each combination of six6 genotype and ocean age across historical (1969–1976) and contemporary (2014–2016; 2019–2022) time periods. [file EVA-19-e70300-s002.docx]

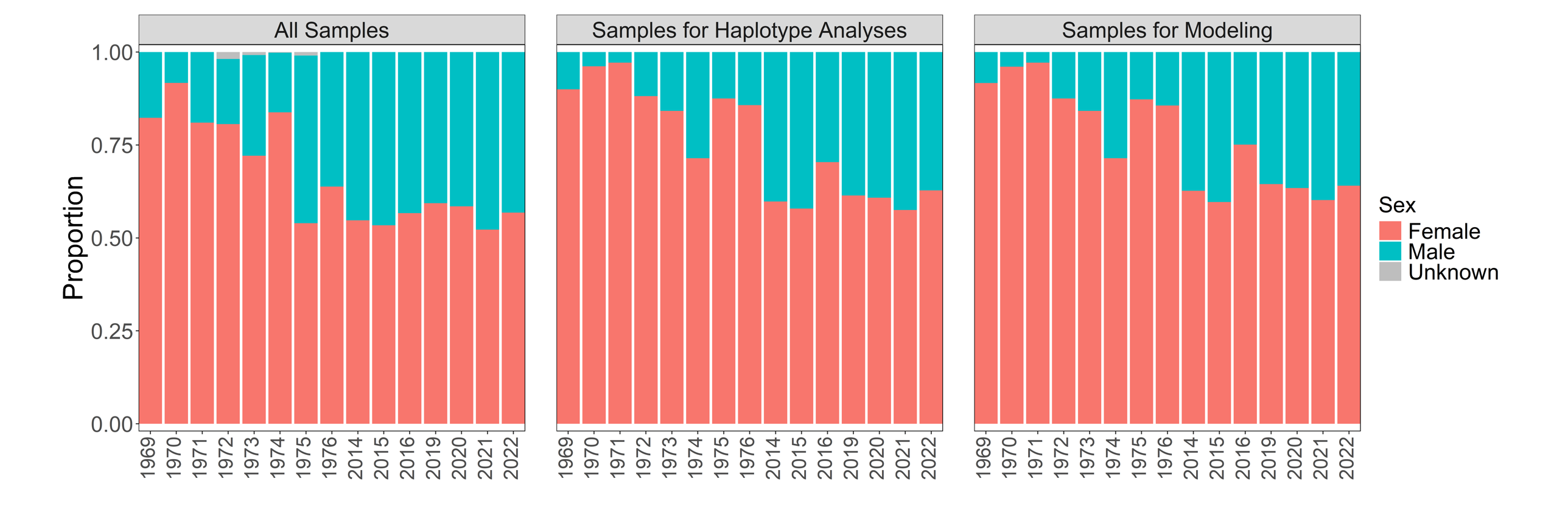


Figure S1. Sex ratio of all collected samples, samples used for haplotype analyses, and samples used for modeling in each spawn year.





Figure S2. *Six6* haplotype and genotype frequencies of females in Dworshak National Fish Hatchery steelhead broodstocks across historical (1969–1976) and contemporary (2014–2016; 2019–2022) time periods. (a) *Six6* haplotype frequencies by year. Years with statistically significant differences in haplotype frequencies from 1969 are denoted above the bars—† represents differences in only the long haplotype, ‡ denotes differences in both long and short haplotypes, and * signifies differences in short, long, and other haplotypes. (b) *Six6* genotype frequencies by year.


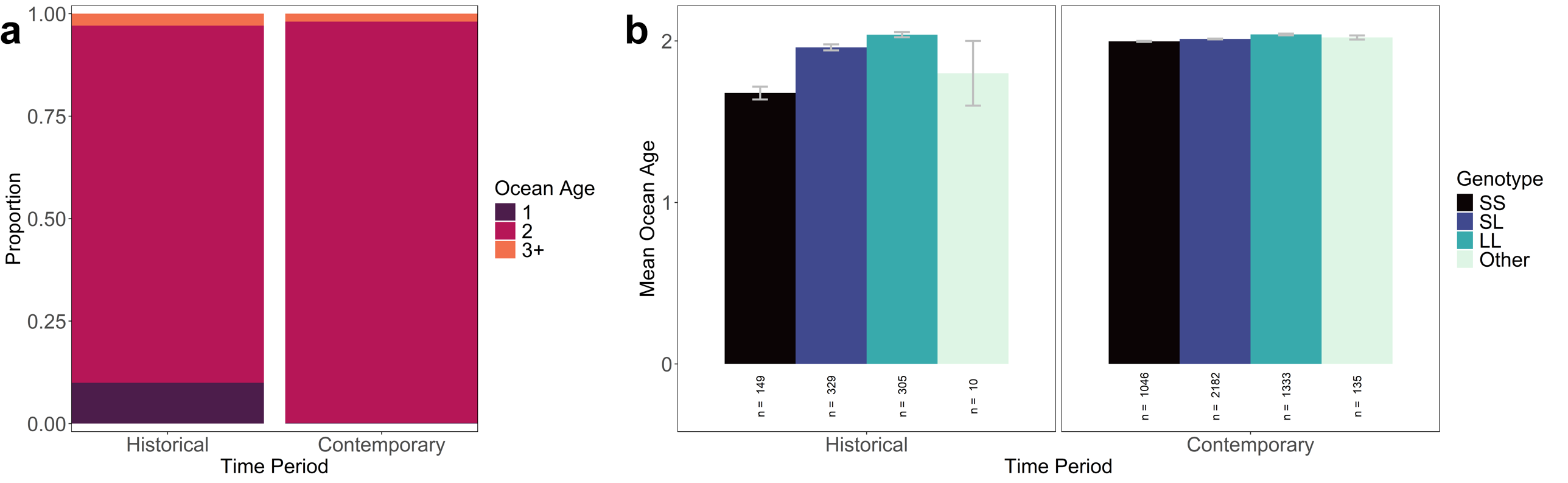


Figure S3. Overall ocean age composition and mean ocean age by *six6* genotype of females in Dworshak National Fish Hatchery steelhead broodstocks across historical (1969–1976) and contemporary (2014–2016; 2019–2022) time periods. (a) Proportion of individuals in each ocean age class. (b) Mean ocean age by *six6* genotype class. Error bars represent standard error.





Figure S4. Predicted mean length for females of each *six6* genotype across historical (1969–1976) and contemporary (2014–2016; 2019–2022) time periods. Error bars represent 95% confidence intervals for each estimated marginal mean.


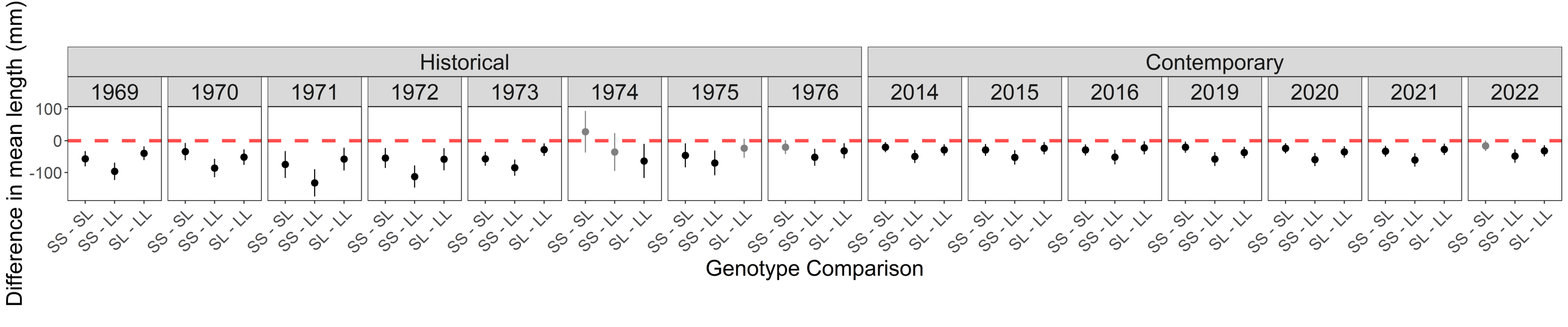


Figure S5. Contrasts of predicted length for females between genotype categories within each year. Error bars represent 95% confidence intervals for each contrast, and statistical significance is denoted by color. Zero is denoted by the dashed red line. Contrasts that significantly differ from zero are black, and contrasts that overlap with zero are gray.


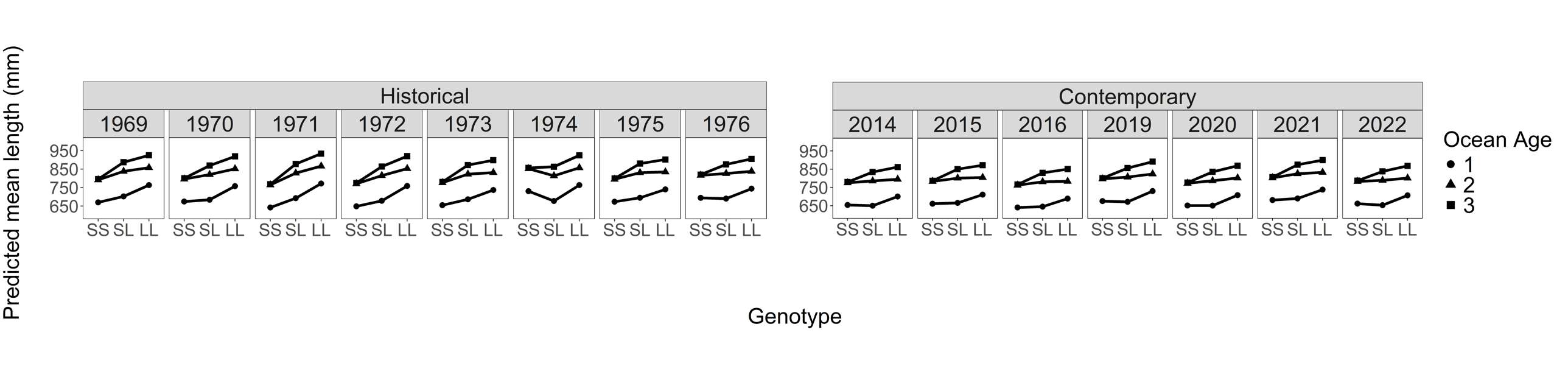


Figure S6. Predicted mean length for females of each combination of *six6* genotype and ocean age across historical (1969–1976) and contemporary (2014–2016; 2019–2022) time periods.
